# Supplementary figures and images for: Protective effect of Mediterranean-type glucose-6-phosphate dehydrogenase deficiency against Plasmodium vivax malaria
Source: eLife. 2021 Feb 5;10:e62448. doi: 10.7554/eLife.62448 (PMC7884069; doi:10.7554/eLife.62448)

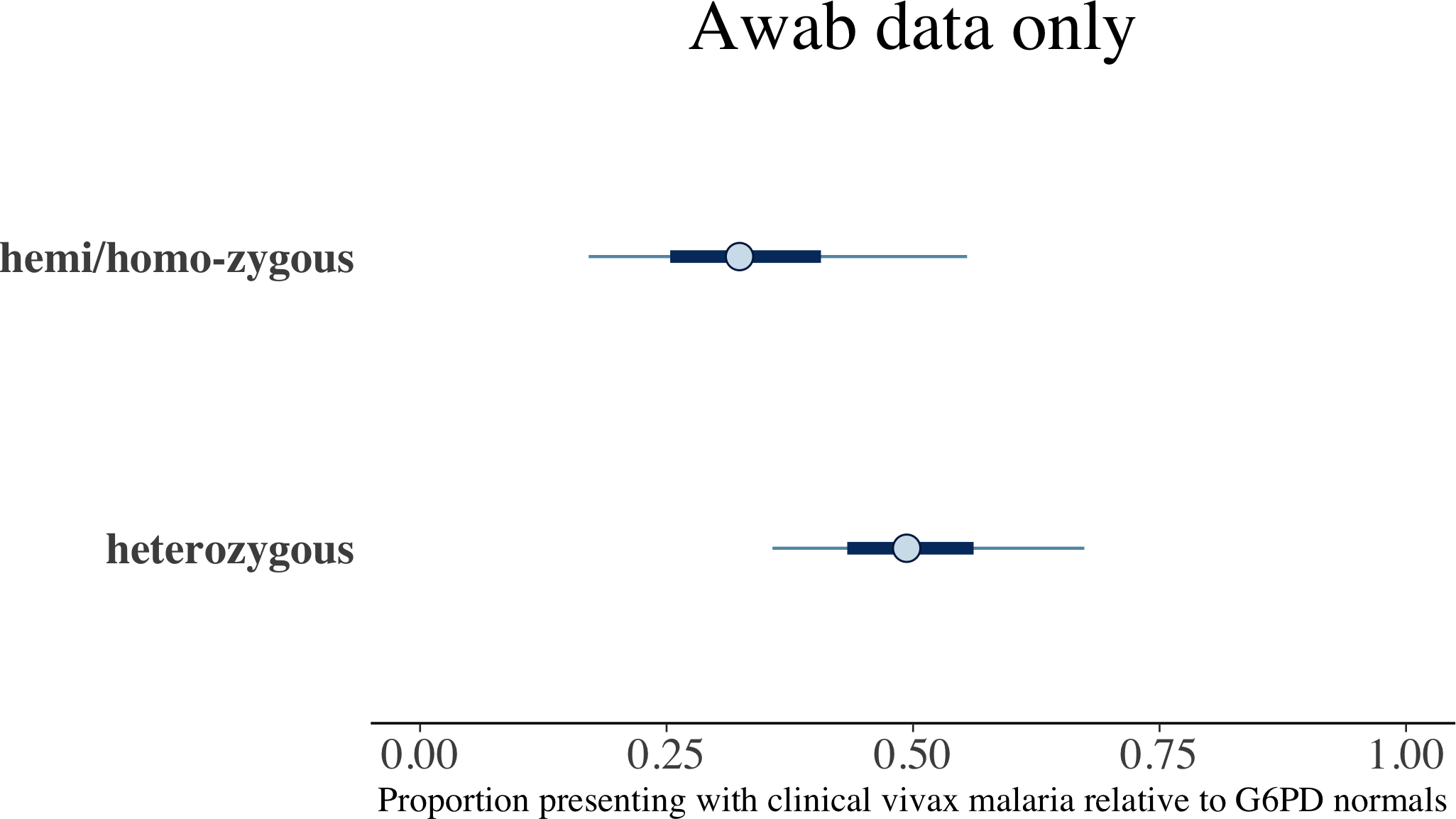

Supplement: Source code 1. [file elife-62448-code1.zip › Published_Code_Data/Meta_Analysis_G6PDMed_files/figure-latex/Awab_results-1.png]

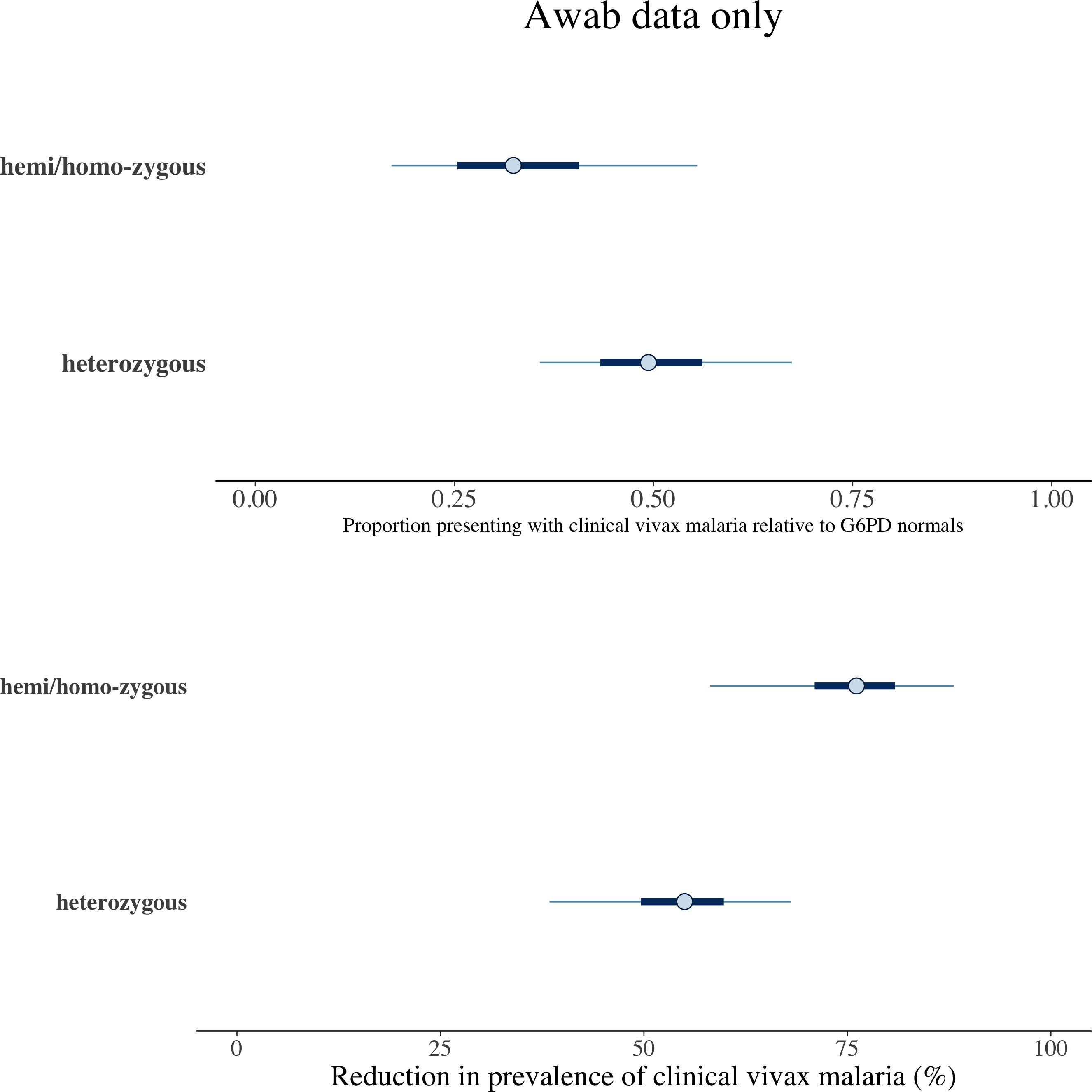

Supplement: Source code 1. [file elife-62448-code1.zip › Published_Code_Data/Meta_Analysis_G6PDMed_files/figure-latex/meta_and_awab-1.png]

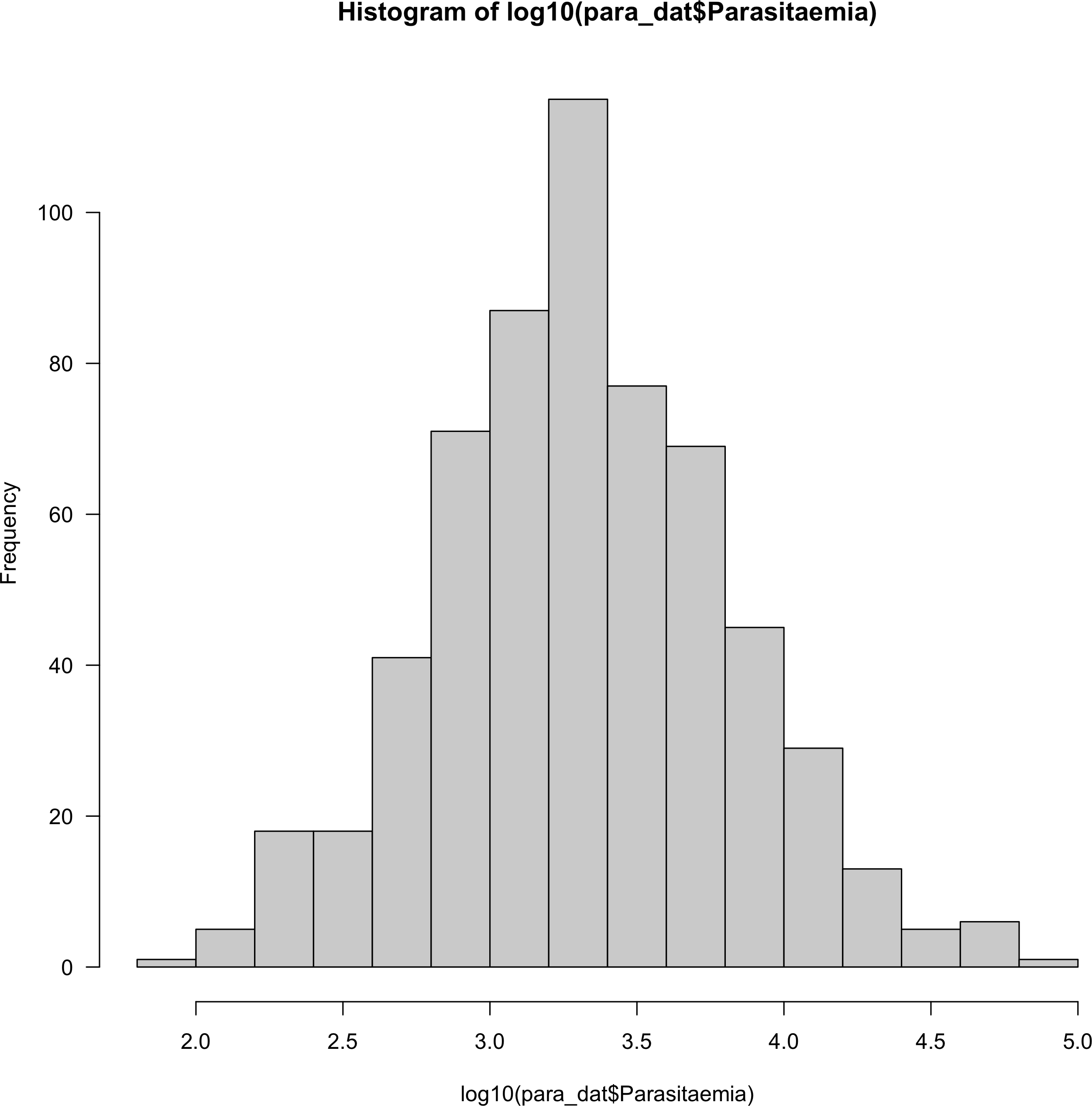

Supplement: Source code 1. [file elife-62448-code1.zip › Published_Code_Data/Meta_Analysis_G6PDMed_files/figure-latex/unnamed-chunk-1-1.png]

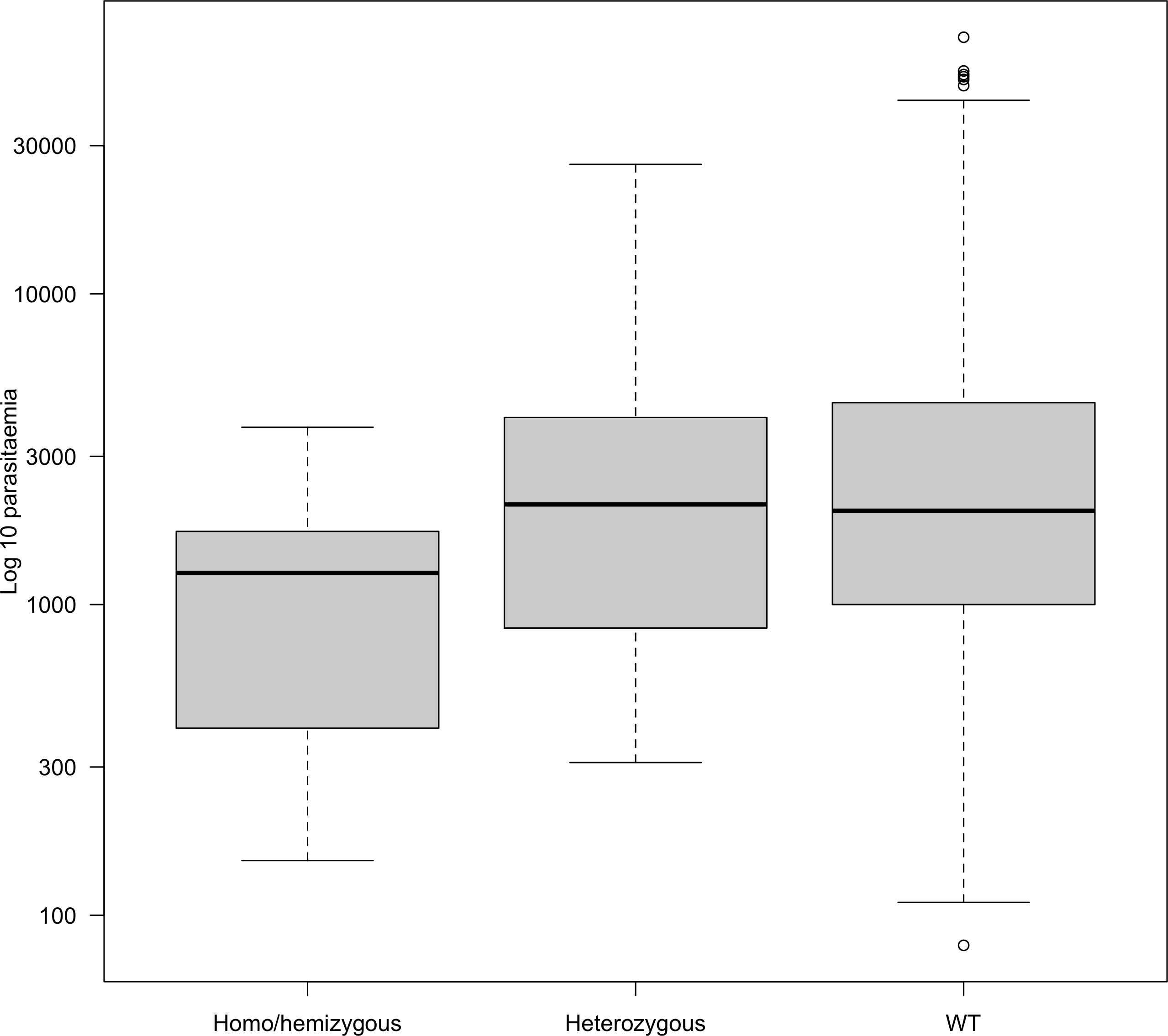

Supplement: Source code 1. [file elife-62448-code1.zip › Published_Code_Data/Meta_Analysis_G6PDMed_files/figure-latex/unnamed-chunk-1-2.png]

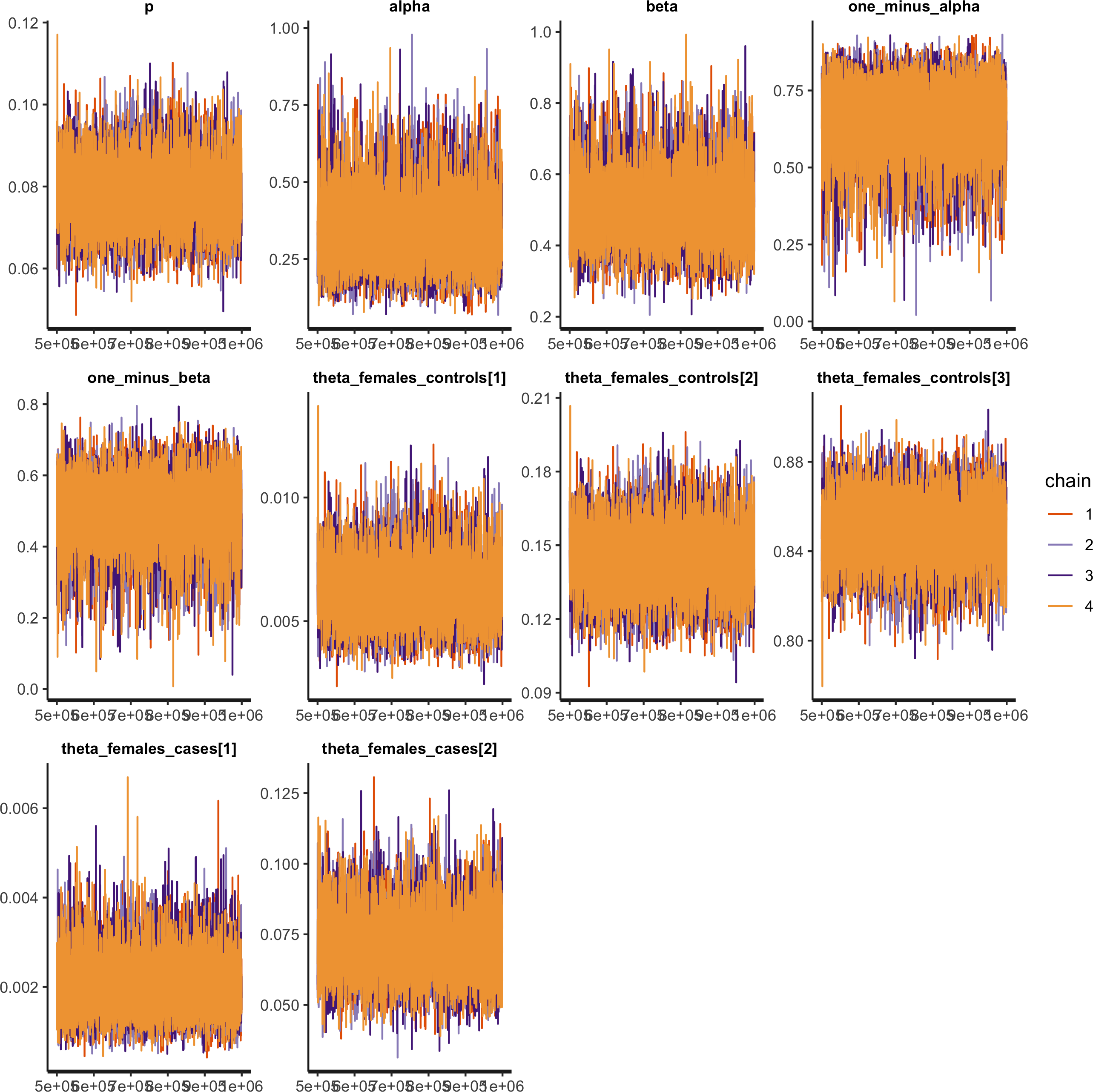

Supplement: Source code 1. [file elife-62448-code1.zip › Published_Code_Data/Meta_Analysis_G6PDMed_files/figure-latex/unnamed-chunk-7-1.png]

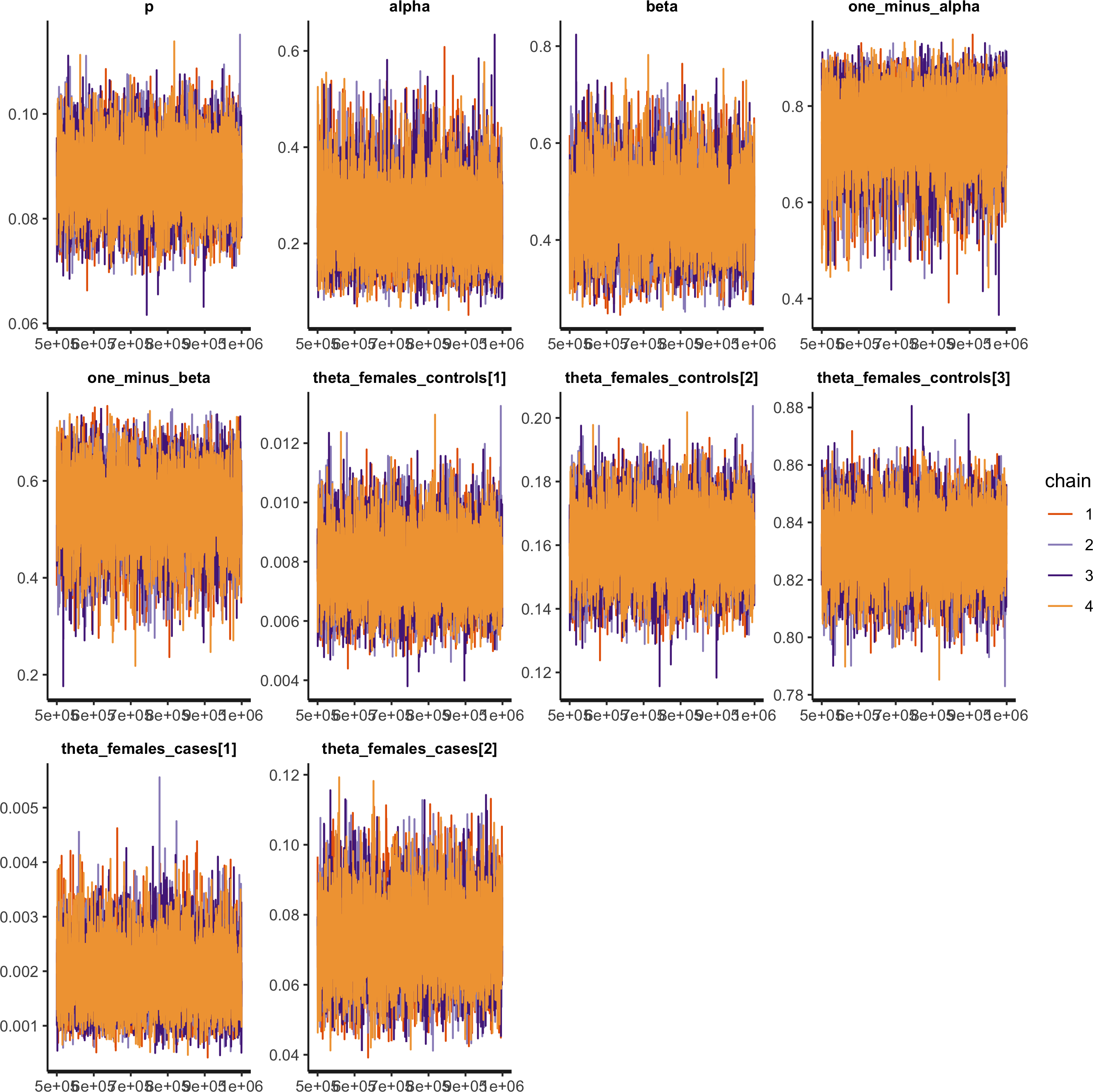

Supplement: Source code 1. [file elife-62448-code1.zip › Published_Code_Data/Meta_Analysis_G6PDMed_files/figure-latex/unnamed-chunk-7-2.png]

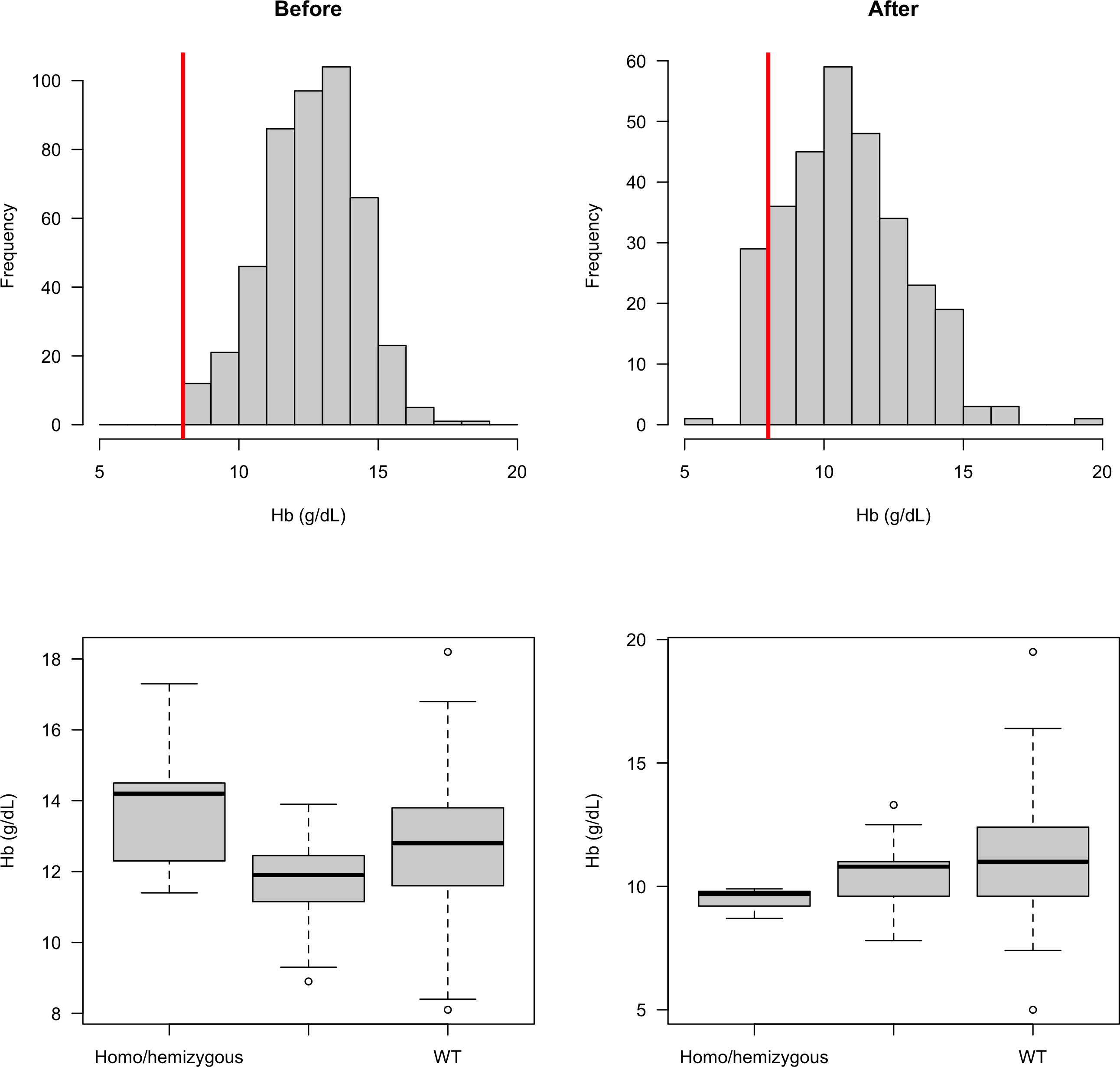

Supplement: Source code 1. [file elife-62448-code1.zip › Published_Code_Data/Meta_Analysis_G6PDMed_files/figure-latex/unnamed-chunk-2-1.png]

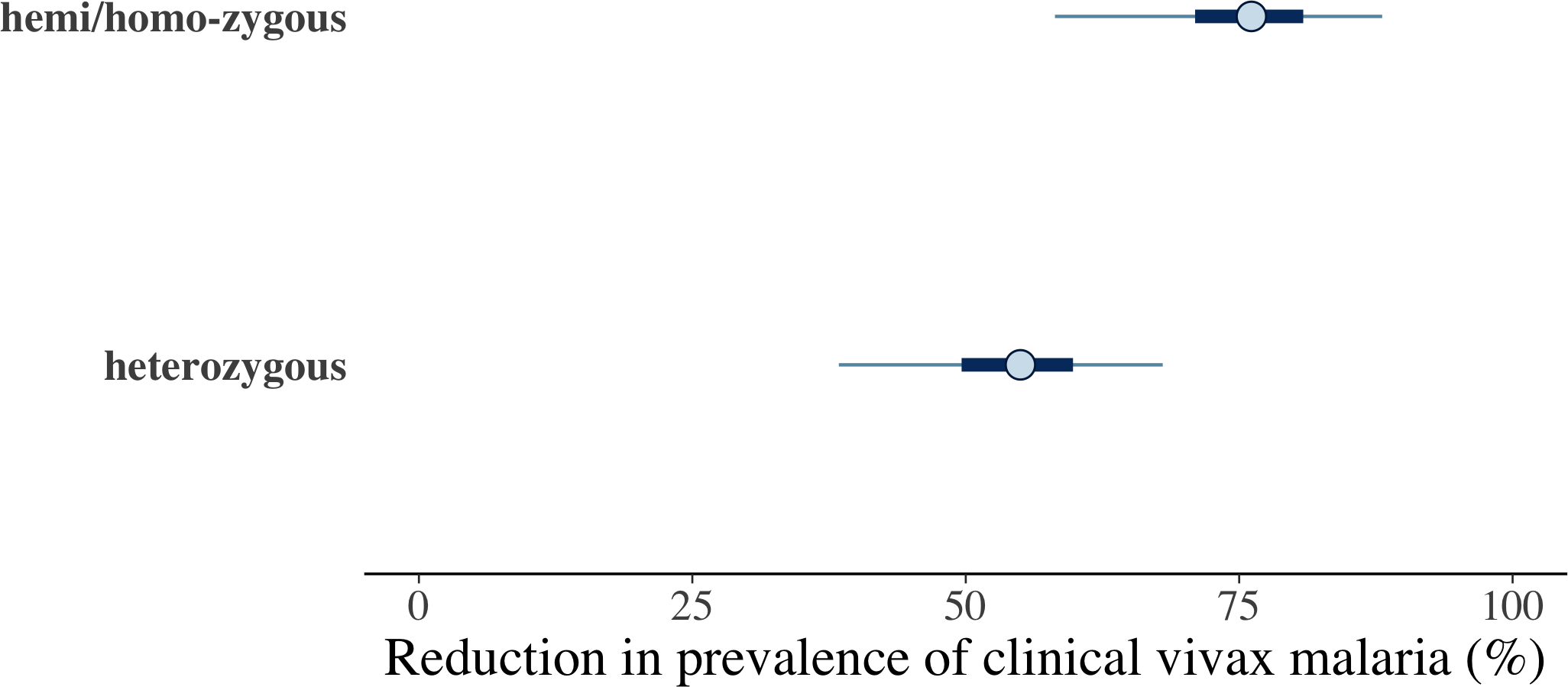

Supplement: Source code 1. [file elife-62448-code1.zip › Published_Code_Data/Meta_Analysis_G6PDMed_files/figure-latex/metaanalysis_results-1.png]
